# Evaluation of a novel closed-loop fluid-administration system based on dynamic predictors of fluid responsiveness: an *in silico *simulation study

**DOI:** 10.1186/cc10562

**Published:** 2011-11-23

**Authors:** Joseph Rinehart, Brenton Alexander, Yannick Le Manach, Christoph K Hofer, Benoit Tavernier, Zeev N Kain, Maxime Cannesson

**Affiliations:** 1 Department of Anesthesiology & Perioperative Care, University of California, Irvine 101 S City Drive, Orange, CA 92868, USA; 2 Department of Anesthesiology and Critical Care Medicine, Centre Hospitalier Universitaire Pitié-Salpêtrière, Paris, France; 3 Centre for Statistics in Medicine, Wolfson College, University of Oxford, Oxford, UK; 4 Institute of Anesthesiology and Intensive Care Medicine, Triemli City Hospital, Zurich, Switzerland; 5 Department of Anesthesiology and Critical Care Medicine, Centre Hospitalier Universitaire de Lille, Lille, France

## Abstract

**Introduction:**

Dynamic predictors of fluid responsiveness have made automated management of fluid resuscitation more practical. We present initial simulation data for a novel closed-loop fluid-management algorithm (LIR, Learning Intravenous Resuscitator).

**Methods:**

The performance of the closed-loop algorithm was tested in three phases by using a patient simulator including a pulse-pressure variation output. In the first phase, LIR was tested in three different hemorrhage scenarios and compared with no management. In the second phase, we compared LIR with 20 practicing anesthesiologists for the management of a simulated hemorrhage scenario. In the third phase, LIR was tested under conditions of noise and artifact in the dynamic predictor.

**Results:**

In the first phase, we observed a significant difference between the unmanaged and the LIR groups in moderate to large hemorrhages in heart rate (76 ± 8 versus 141 ± 29 beats/min), mean arterial pressure (91 ± 6 versus 59 ± 26 mm Hg), and cardiac output (CO; (6.4 ± 0.9 versus 3.2 ± 1.8 L/min) (*P *< 0.005 for all comparisons). In the second phase, LIR intervened significantly earlier than the practitioners (16.0 ± 1.3 minutes versus 21.5 ± 5.6 minutes; *P *< 0.05) and gave more total fluid (2,675 ± 244 ml versus 1,968 ± 644 ml; *P *< 0.05). The mean CO was higher in the LIR group than in the practitioner group (5.9 ± 0.2 versus 5.2 ± 0.6 L/min; *P *< 0.05). Finally, in the third phase, despite the addition of noise to the pulse-pressure variation value, no significant difference was found across conditions in mean, final, or minimum CO.

**Conclusion:**

These data demonstrate that LIR is an effective volumetric resuscitator in simulated hemorrhage scenarios and improved physician management of the simulated hemorrhages.

## Introduction

Automation is ubiquitous in modern life but historically has found limited application in medical care. However, recent interest has led to the rapid growth of research in automated controllers in areas ranging from glucose management to sedation to mechanical ventilation (1-3). Closed-loop (automated) controllers have been shown to manage patients safely and more consistently than clinicians for myriad applications [1-3], but a key requirement for proper function is reliable feedback data from systems being controlled. In the case of fluid responsiveness, historical measures like urine output, central venous pressure, and pulmonary capillary wedge pressure are very poor predictors and thus unsuitable for use in a closed-loop system [4].

Fortunately, great progress has been made in two areas that now make automated fluid management practical [5]. The first is the description and characterization of the dynamic predictors of fluid responsiveness. Parameters like pulse-pressure variation (PPV), stroke volume variation (SVV), or respiratory variations in the plethysmographic waveform amplitude (ΔPOP) allow a reliable determination of whether a mechanically ventilated patient is likely to respond to a fluid bolus with a subsequent increase in cardiac output (CO) [6-8]. This strong predictive relation can be used to guide resuscitation, and fluid therapy based on the dynamic predictors has suggested improved outcomes in recently published prospective trials [8,9].

The second area is the rapid advancement in monitoring technology; noninvasive and increasingly accurate monitors can provide vital-signs data previously available only through invasive approaches.

As one of the first steps in the development of an automation algorithm is testing under simulation [10-15], we present data for a novel closed-loop fluid-management algorithm (LIR: Learning Intravenous Resuscitator) in simulation studies using heart rate (HR), mean arterial pressure (MAP), CO, and PPV as the input variables. The goals of the present study were (a) to assess the performance of LIR in a spectrum of bleeding scenarios, (b) to compare automated fluid management by LIR with standard fluid management by practitioners in simulation cases, and finally (c) specifically to challenge the reliance of LIR on the accuracy of PPV for effective resuscitation.

## Materials and methods

The study was performed during October and November of 2010 and February of 2011 at the UCI Medical Center in Orange, California. IRB exemption was obtained for the work done with faculty and residents.

### Closed-loop algorithm design

The LIR algorithm is an adaptive controller that incorporates data from previous clinical trials in its decision engine. It monitors a variety of patient hemodynamic parameters (CO, dynamic predictors like PPV and stroke volume variation, heart rate, and blood pressure) and uses this information to control fluid administration. The design of the algorithm is described in the following sections.

### Database construction for use in the controller

A dataset of 414 patients, which contained hemodynamic parameters before and after a 500-ml bolus of hetastarch 6% or modified fluid gelatin given over a 10- to 20-minute period, was used to derive population-based formulas for guiding fluid therapy based on PPV and CO. This population, the method used, and the way CO was measured have been described elsewhere in detail [16]. Institutional review board (Comité de Protection des Personnes Hospices Civils de Lyon, Lyon, France, Comité de Protection des Personnes Paris-Ile de France, France, Comité de Protection des Personnes Nord Ouest, Lille, France, and Institutional Review Board Triemli City Hospital, Zurich, Switzerland) approvals were obtained. As described previously, patients were included either as part of clinical trials or as part of routine clinical care [16]. CO was measured in all patients (a) by thermodilution via a pulmonary artery catheter (PAC; Swan-Ganz catheter, 7.5F; Edwards LifeScience, Irvine, CA, USA), or (b) by the pulse-contour method by using a 4F thermistor-tipped arterial catheter (Pulsiocath thermodilution catheter) inserted into the left femoral artery and connected to a stand-alone PiCCOplus or PiCCO_2 _monitor (Pulsion Medical Systems, Munchen, Germany); or (c) via transesophageal echocardiography [16].

### Controller characteristics

**Figure 1:**
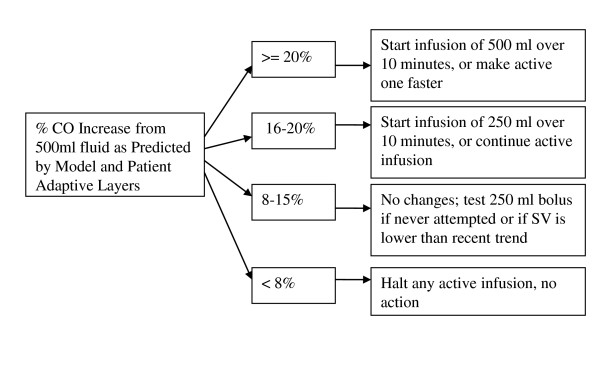
**Rule-based component of the controller algorithm**. The controller uses patient hemodynamic parameters (primarily pulse-pressure variation, but also cardiac output, mean arterial pressure, and heart rate) that are compared with the dataset and a probability of positive response assigned based on the population data. This probability and the hemodynamic data are then fed into the rule-based component of the controller. CO, Cardiac output.

The controller uses the resulting database to calculate when a patient is likely to respond to a fluid bolus and with what degree of increase in CO. Patient hemodynamic parameters (primarily PPV, but also CO, mean arterial pressure (MAP), and HR) are compared with the dataset, and a probability of positive response is assigned based on the population data. This probability and the hemodynamic data are then modified based on previous patient responses to fluid administration and deviations from the model predictions. The core rule-based component of the system is shown in Figure 1. The net expected percentage increase in CO predicted by the previous layers is used to direct therapy. If little to no increase in CO is expected, no fluid bolus is given; if an infusion is already being given, it is halted. Conversely, if the expected percentage increase is high, a fluid bolus is started (or quickened). In the middle range--the portion corresponding to the recently described "gray zone" [16]--no actions are taken, and if a bolus is already running, it will be allowed to continue. Under some circumstances (for example, a downward trend in CO), a test bolus may be given in this middle range to assess the patient response [17].

As implied earlier, the algorithm was designed to be adaptive. It uses a bolus-based approach to fluid management (as opposed to continuous ongoing infusion) to allow the algorithm to analyze the efficacy of its interventions and to modify its own activity. As successive boluses are given, the hemodynamic data before and after each bolus are recorded by the algorithm. These data are used to modify population predictions with regard to the current patient, as well as the boundaries of the tree shown in Figure 1. In the decision tree, for example, if a patient falls into the indeterminate range of the decision tree and a test bolus is given with no improvement in CO, then the threshold required for a test bolus in the indeterminate zone will be raised by the algorithm. Similarly, if a bolus is given by the system and a much larger increase in CO is noted than was expected, the threshold for future boluses will be lowered.

### Simulator design

Because no commercial simulators currently include dynamic predictors like PPV, we developed one to test the closed-loop algorithm. The design and validation of the simulator is included in Additional file 1. The software was run on a PC during these studies, with simulated patient data displayed on the PC monitor and the participants interacting through the mouse and keyboard.

Participants were able to control infusions (crystalloids or colloids) and administer ephedrine, phenylephrine, epinephrine, or fentanyl. As ventilator parameters (mode, rate, tidal volume) were not of specific interest for this study, they were excluded from the participant controls. All the numeric parameters available to the LIR algorithm (HR, MAP, CO, and PPV), in addition to others (SBP, DBP, SPO_2_, CO_2_), were shown on the participants' monitor during the simulation. Blood products were not available for infusion; LIR was not designed to determine the appropriate fluid to administer (this would be at the discretion of the supervising physician), but only the appropriate volumes. As such, the decision was made to allow only crystalloids and colloids to keep the participant and LIR conditions comparable.

### Study protocol

As stated, the goals of this preliminary study were to (a) to evaluate the performance of LIR in simulated hemorrhage scenarios, (b) to compare fluid management by LIR with standard fluid management by practitioners in those scenarios, and (c) specifically to test the controller in the absence of highly accurate PPV data. These objectives were split into phases and are detailed in Table 1 for reference.

**Table 1:** Study phases and groups

Phase	Scenario(s)	PPV condition	Management
Phase 1: Testing of	Massive hemorrhage	Accurate PPV	No management
stability (2.5-hour simulation)	Moderate hemorrhage		LIR management
	Mild hemorrhage		
			

Phase 2: Comparison with	Massive hemorrhage	Accurate PPV	No management
practitioner management (1-hour simulation)			Practitioner management
			Practitioner meds/LIR Management
			LIR management

Phase 3: noise and artifact	Mild hemorrhage	Accurate	LIR management
tolerance (2-hour simulation)		Biased	
		Fluctuating	
		biased and fluctuating	

LIR, Learning Intravenous Resuscitator; PPV, pulse-pressure variation.

#### Phase 1: Basic evaluation of the controller in hemorrhage scenarios

The first phase evaluated the basic efficacy of the controller and its ability to manage fluids during hemorrhage scenarios. The simulator was run on one PC with vital signs (HR, systolic and diastolic blood pressure, CO, and PPV) being recorded over the network by a separate PC running the LIR algorithm. Interventions (in the form of fluid boluses) were communicated back to the simulator over the network. Three simulation scenarios were run during this phase: (a) mild hemorrhage of 500 ml over a 1.5-hour period; (b) moderate hemorrhage of 1,500 ml over a 1.5-hour period, and (c) massive hemorrhage of 2,000 ml over a 20-minute period. Patient height, weight, baseline HR, and baseline SBP/DBP were randomized within preset ranges (Table 2) In each scenario, bleeding began 30 minutes after the start of the simulation, and the scenario ran for 2.5 hours before finishing. For each scenario, two management groups were tested; 20 trials were performed with no hemodynamic management (to demonstrate the effects of the simulated hemorrhage scenarios on hemodynamic parameters), and 20 additional trials received crystalloid infusions managed by the LIR algorithm. Both groups received a steady infusion rate of 200 ml/hr of crystalloid.

**Table 2:** Baseline parameter ranges for study phases

	Phase 1		Phase 2		Phase 3	
	Min	Max	Min	Max	Min	Max
Weight (kg)	60	100	70	85	70	85
Height (in)	62	72	65	68	65	68
HR (beats/min)	55	85	65	75	65	75
SBP (mm Hg)	105	145	110	130	110	130
DBP (mmHg)	60	90	70	80	70	80
LVEDV (ml)	130	150	130	150	130	150
LVESV (ml)	42	58	42	58	42	58
						

DBP, diastolic blood pressure; LVEDV, left ventricular end-diastolic blood pressure; LVESV, left ventricular end-systolic volume; SBP, systolic blood pressure.

#### Phase 2: Closed-loop system versus practitioner management during simulated hemorrhage scenarios

The second phase of testing compared the fluid management of practitioners with that of the LIR algorithm in simulated hemorrhage cases. After IRB exemption was obtained, 20 academic anesthesiologists and residents were asked to manage fluids and medications for a 1-hour simulated case of massive hemorrhage (2,000 ml blood loss over 20 minutes). The subjects were given the following history:

"You are taking over management of an otherwise healthy 40-year-old woman who is having a pelvic tumor debulking. The surgery started 1 hour ago, and anesthetic management has been uncomplicated. Baseline chemistries were within normal ranges, and the starting hematocrit was 39%."

Subjects were allowed to ask questions, and responses were standardized from a preset list of available information. Simulator output was presented on the PC graphically similarly to the monitors used in the operating rooms, showing HR, blood pressure, pulse oximetry, CO, and PPV. The hemorrhage began 15 minutes into the simulation and continued for 20 minutes. Five minutes after the hemorrhage began, the subject managing the scenario was told, "The surgical team tells you they're losing a lot of blood." Five minutes after the hemorrhage ended, they were told that the bleeding seemed to be under control. The practitioners could give crystalloid, ephedrine, 10 mg, phenylephrine, 100 mg, or fentanyl, 50 μg, during the management of the simulated patient. One week later, the subjects repeated the simulation, but this time the rate of crystalloid infusion was secretly managed by the closed-loop system while practitioner fluid administrations were ignored and only the medications affected the simulator. The simulation was also run 20 times with only LIR managing crystalloids and 20 times with no management. The same clinical scenario was run for each practitioner, and the baseline characteristics of the simulated patient were again randomized by the computer within a small range of preset parameters (Table 2). The range of randomization was narrowed for this phase so that differences in management would be highlighted, as opposed to differences in the simulation.

#### Phase 3: Testing the controller's dependence on accurate PPV

For the third phase of testing, the simulator was modified such that PPV was no longer as accurate a predictor of fluid responsiveness. Four conditions were run during this phase of testing:

1. Accurate PPV: the PPV was perfectly predictive of the response to fluid;

2. Biased PPV: the PPV value was constantly biased ± 5% (absolute value) from the true value throughout the entire scenario;

3. Fluctuating PPV: the PPV value fluctuated randomly from ± 5% (absolute value) of the true value at random during the scenario; and

4. Biased and fluctuating PPV: the PPV had both a steady bias within ± 5% (absolute value) and an additional fluctuating component of ± 5% (absolute value). Thus, in the final condition, the PPV reported by the simulator may have been up 10% different from the true value; a "true" PPV of 15% might have been reported as anywhere from 5% to 25%). The closed loop was then used to manage crystalloid infusions for a 2-hour, 1,000-ml blood-loss scenario under each of these four conditions, with bleeding starting 30 minutes into the simulation. A longer and slower hemorrhage scenario than was used in Phase 2 was intentionally simulated for this phase to accentuate the differences in the accuracy of the PPV across groups; a massive hemorrhage might have hidden small differences.

### Statistical analysis

Data are presented as mean ± SD. For Phase 1, data between the no intervention group and the LIR management group were compared by using a Mann-Whitney test. For Phases 2 and 3, data were compared by using analysis of variance for repeated measurements (ANOVA). If significant differences were found, *post hoc *testing was performed by using Tukey's honest significant difference. A *P *value < 0.05 was considered statistically significant. All statistic analysis was performed by using SPSS 13.0 for Windows, SPSS, Chicago, IL, USA.

## Results

### Phase 1

#### Basic evaluation of the controller in hemorrhage scenarios

Mean CO, HR, and blood pressure were similar across groups at baseline (6.5 ± 1.1 L/min, 71 ± 9 beats/min, and 90 ± 8 mm Hg, respectively). In the massive hemorrhage scenario, a significant difference was noted between the unmanaged and the closed-loop managed conditions in HR, MAP, and CO throughout the case and at the end of the simulation (Table 3). Likewise, in the moderate-hemorrhage scenario, a significant difference was found between the unmanaged and the closed-loop managed groups in HR, MAP, and CO throughout the case and at the end of the simulation (Table 3). No significant difference existed between management groups in the mild-hemorrhage scenario (Table 3). The closed-loop administered fluid before clinical evidence of hemorrhage was apparent from CO, HR, or MAP. In the massive-hemorrhage scenario, for example, the average time to show a 10% decrease from baseline CO and MAP was 14 ± 4 minutes and 16 ± 3 minutes, respectively, from the start of the hemorrhage, and HR increased to 10% over baseline at 13 ± 3 minutes. LIR, meanwhile, began administering fluid on average at 7 ± 2 minutes. PPV was the earliest indicator of hemorrhage, increasing to 10% within 7 ± 2 minutes and 15% within 8 ± 2 minutes in this scenario.

**Table 3:** Final hemodynamic parameters in Phase 1 groups

	No intervention (*n *= 20)	Closed-loop management (*n *= 20)	*P *value
Massive hemorrhage			
Fluid given (ml)	300 ± 0	3,420 ± 117	
HR (beats/min)	141 ± 29	76 ± 8	< 0.001
MAP (mm Hg)	59 ± 26	91 ± 6	< 0.001
CO (L/min)	3.2 ± 1.8	6.4 ± 0.9	< 0.001
Moderate hemorrhage			
Fluid given (ml)	300 ± 0	1,543 ± 54	
HR (beats/min)	119 ± 32	73 ± 9	< 0.001
MAP (mm Hg)	76 ± 10	88 ± 7	< 0.005
CO (L/min)	5.0 ± 1.1	6.9 ± 0.8	< 0.001
Mild hemorrhage			
Fluid given (ml)	300 ± 0	653 ± 44	
HR (beats/min)	77 ± 10	72 ± 9	0.08
MAP (mm Hg)	85 ± 7	87 ± 8.8	0.3
CO (L/min)	6.6 ± 1.0	6.5 ± 1.0	0.73

Data are presented as mean ± SD. CO, cardiac output; HR, heart rate; MAP, mean arterial pressure.

### Phase 2

#### Closed-loop system versus practitioner management during simulated-hemorrhage scenarios

**Figure 2:**
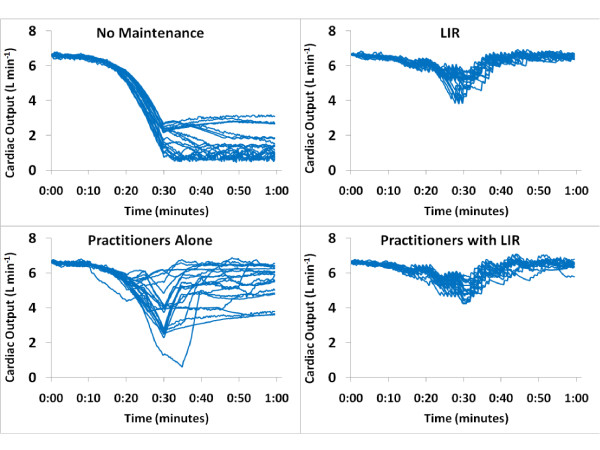
**Cardiac output in Phase 2 groups; closed-loop system versus practitioner management during a simulated hemorrhage scenario**. Each line represents a single case. Once the hemorrhage began, the LIR-managed groups intervened significantly earlier than the practitioner group and gave more total fluid. The mean, minimum, and final cardiac output was higher in both LIR-managed groups than in the practitioner group, and the coefficient of variance was lower. LIR, Learning Intravenous Resuscitator.

**Figure 3:**
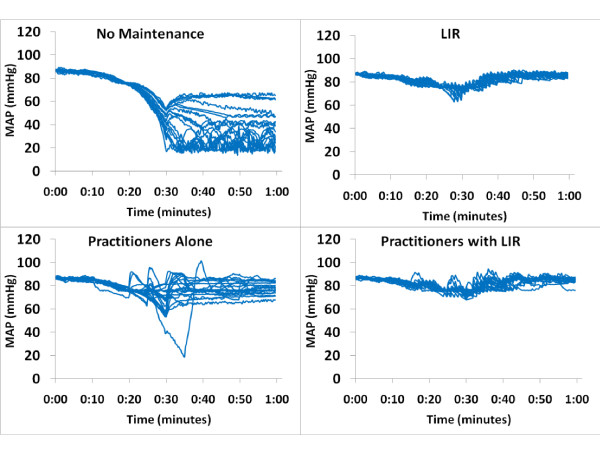
**Mean arterial pressure in Phase 2 groups: closed-loop system versus practitioner management during a simulated hemorrhage scenario**. Each line represents a single case. We observed no difference in mean arterial pressure between intervention groups, but all were significantly higher than those in the unmanaged group.

Eleven residents and nine attending anesthesiologists participated in the simulation. The residents were PGY-2 to PGY-4, aged 28 to 34 years, with seven men and four women. Attendings consisted of five men and four women, and had been practicing a median of 5 years (range, 1 to 15 years). Simulated patient HR, MAP, and CO values were similar at baseline across all four management groups (6.5 ± 0.2 L/min, 68 ± 2 beats/min, and 85 ± 2 mm Hg, respectively). Once the hemorrhage began, the LIR-managed groups intervened significantly earlier than the practitioner group and gave more total fluid (Table 4). The mean, minimum, and final CO values were higher in both LIR-managed groups than in the practitioners group (Figure 2), and the coefficient of variance was lower (Table 4 and Figure 2). No difference in MAP was found between intervention groups, but all were significantly higher than the unmanaged group (Figure 3). No significant difference appeared between attending and resident performance in final CO, HR, or MAP values in the practitioner-management group (4.5 ± 1.2 L/min, 95 ± 13 beats/min, and 76 ± 10 mm Hg for attendings, and 4.9 ± 1.7 L/min, 91 ± 28 beats/min, and 75 ± 19 mm Hg for residents). Finally, a significant reduction in vasopressor use was noted in the Practitioners with LIR group versus the Practitioners group (Table 5).

**Table 4:** Fluid management: anesthesiologists versus closed loop

	No management (1)	Anesthesiologist managed (2)	Anesthesiologist, pressors; closed-loop, fluids (3)	Closed-loop managed (4)
Time window until the application of first bolus from start of hemorrhage (min)	-	21.5 ± 5.6^a^	15.6 ± 1.1	16.0 ± 1.3
Total fluid given (ml)	-	1,968 + 644^a^	2,875 ± 275	2,675 ± 244
Mean arterial pressure (mm Hg)	61 ± 6.9	76 ± 4.2	79 ± 2.0	79 + 1.1
Mean cardiac output (L/min)	3.8 ± 0.4	5.2 ± 0.6^a^	5.8 ± 0.2^b^	5.9 ± 0.2^b^
Minimum cardiac output (L/min)	1.4 ± 0.8	3.6 ± 1.3^a^	4.8 ± 0.5^b^	4.8 ± 0.4^b^
Final cardiac output (L/min)	1.7 ± 0.9	4.8 ± 1.5^a^	5.6 ± 0.5^b^	5.7 ± 0.4^b^
Cardiac output during case, coefficient of variance (%)	89 ± 29	36.7 ± 23^a^	16.6 ± 9^b^	16.3 ± 8^b^

*n *= 20 in each group. Data are reported as mean ± standard deviation. ^a^*P *< 0.05 versus groups 2, 3, and 4. ^b^*P *< 0.05 versus groups 1 and 4.

**Table 5:** Ephedrine and phenylephrine use in Phase 2 of the study

	Ephedrine (mg)	Phenylephrine (μg)
No management	0 ± 0	0 ± 0
LIR alone	0 ± 0	0 ± 0
Practitioners alone	0 ± 0	100 ± 132^a^
Practitioners with LIR	0.3 ± 1.1^b^	40 ± 94^b^

Data are presented as mean ± SD. a *P *< 0.05 for practitioners alone versus LIR alone. ^b^*P *< 0.05 for practitioners with LIR versus practitioners alone. LIR, Learning Intravenous Resuscitator.

### Phase 3. Testing the controller's dependence on accurate PPV

Baseline CO, HR, and MAP values across all trial conditions were similar at baseline in this phase (6.6 ± 0.4 L/min, 70 ± 1 beats/min, and 87 ± 2 mm Hg, respectively). No significant difference was found in mean, minimum, or final CO across PPV conditions, regardless of the type of artifact induced in the PPV signal (Table 6). Time to first fluid bolus and total volume infused were likewise nonsignificant. Although the coefficient of variance of the CO during the case did increase from 5.4% ± 1.3% in the perfect PPV condition to 7.0% ± 3.2% in the biased and fluctuating PPV condition, this difference was also not significant (*P *= 0.06).

**Table 6:** Closed-Loop Fluid Management - Uncertain PPV Conditions

	Perfect PPV	Biased PPV	Fluctuating PPV	Biased & Fluctuating PPV
**First Bolus (min)**	52.6 ± 0.9	53.4 ± 1.4	51.9 ± 4	52.6 ± 3.9
**Total Fluid Given (ml)**	2476 ± 85	2466 ± 80	2428 ± 159	2435 ± 131
**Mean Arterial Pressure (mmHg)**	82.1 ± 0.7	82.1 ± 0.6	82 ± 1.2	82 ± 1.6
**Mean Cardiac Output (L/min)**	6.3 ± 0.1	6.3 ± 0.1	6.2 ± 0.2	6.2 ± 0.2
**Minimum Cardiac Output (L/min)**	5.8 ± 0.2	5.9 ± 0.2	5.7 ± 0.3	5.6 ± 0.4
**Final Cardiac Output (L/min)**	6.4 ± 0.1	6.4 ± 0.1	6.4 ± 0.1	6.3 ± 0.2
**Cardiac Output During Case, Coefficient of Variance (%)**	5.4 ± 1.3	5.4 ± 1.1	6.4 ± 2.0	7.0 ± 3.2

*n *= 20 each group. Data are reported as mean +/- standard deviation. p > 0.05 for all comparisons. PPV: pulse pressure variation.

## Discussion

As simulation studies are a standard step in the testing of new controllers [10-15], these data represent the first step in the development of an automated fluid-management system for clinical use. Overall, the results suggest that (a) the LIR algorithm is capable of performing fluid resuscitations, at least in simulation; (b) the controller performs comparably to practitioners in this setting and maintains a higher and more stable CO; and (c) the controller is not dependent on a highly predictive PPV value to function.

The first phase of our simulation study was to show that LIR is capable of adequately resuscitating patients during blood-loss scenarios. Although it was perhaps not surprising that the controller was superior to a low-rate steady crystalloid infusion, this was nevertheless an essential step in the development of the algorithm. One of the key results of this phase is the low standard deviation of the fluid infusions within scenarios; although this is not an engineering test of stability, this suggests at the minimum, consistency of the algorithm in its management. Another important observation from this portion of the study is that LIR did not *over*-resuscitate; the controller stopped administering fluid when the CO began to level off.

In comparison to practitioners in the second phase of the study, LIR detected the need for resuscitation earlier and was more consistent in management. Some individual practitioners maintained CO in the same range that LIR did throughout the case, but a huge interpractitioner variability occurred (Figure 2). This is one of the biggest offerings of closed-loop management: reduction of interpersonal variability and bias in management. Another interesting observation from this phase of the study is that whereas the differences in CO were significant, the differences in MAP between practitioner and LIR groups were not (Figure 3). Still a larger variability in MAP was noted in the practitioner group, but overall, the clinicians maintained MAP closer to baseline than CO. This is consistent with the historical trend in clinical management to focus on blood pressure in hemodynamic management, although evidence now suggests that CO and oxygen delivery should also be central to management.

Although no significant difference was found between attendings and residents in hemodynamic parameters, this study was not designed to look for such differences and was not sufficiently powered toward that end. Interestingly, however, although the means were similar between the two subgroups, the standard deviations for HR, MAP, and CO were much wider in the resident management subgroup versus the attending management subgroup. One would expect that the lesser level of expertise of the resident subgroup might lead to greater variation in management.

Finally, a reduction in vasopressor use by practitioners occurred when LIR was managing fluids (although the practitioners did not know that the closed-loop was actually in use) (Table 5). Interestingly, phenylephrine was chosen almost exclusively over ephedrine during this simulation by the practitioners, despite their having access to both agents at all times. This is just a conjecture, but it seems likely that practitioners chose phenylephrine because the heart rate was already elevated because of hemorrhage when vasopressors were being given.

The final phase of the study demonstrates that LIR is not completely dependent on a highly predictive PPV value to function effectively. In the tests, the greater the uncertainty introduced, the larger the variance in management becomes, but even when the PPV value was off by up to 10% of the true value, the resulting difference in fluid given was less than 75 ml per hour, and represented less than 5% of the total volume administered (Table 4). If closed-loop systems are to be deployed in clinical use, they must be equipped to handle noise, artifact, and the uncertainty present in all clinical work and still operate safely and effectively, and although the controller will certainly need more-definitive tests of robustness, this is a reassuring early result. However, limitations to the use of dynamic parameters of fluid responsiveness will still have to be respected (limitations include spontaneous ventilation, low tidal volume equal to or less than 7 ml/kg, arrhythmia, open-chest procedures, and HR/respiratory rate ratio < 3.6). For this reason, the LIR algorithm is also designed for conducting stroke-volume optimization alone when PPV and SVV are not available [18] and, inversely, to conduct PPV or SVV minimization when stroke volume is not monitored [6]. However, the present study does not test these features, and these results cannot be extrapolated. The same kind of study testing the ability of LIR to handle noise or artifacts for stroke-volume maximization alone or PPV/SVV minimization alone is warranted.

The same patient database was used in the design of both the LIR algorithm and the PPV portion of the simulator with which it was tested, possibly creating a bias that favored the performance of the algorithm. This is a valid concern, but one that was both unavoidable and in part addressed in the third phase of the study. This was unavoidable because both the algorithm and the simulator were designed, to the best of the authors' skill, by using the best available information on how PPV predicts response to fluid in human subjects (see Additional file 1). Because of this, and regardless of how it had actually been accomplished, the systems would have been built by using the same underlying principles governing the dynamic predictors or else, by definition, one (or both) would have included inferior operational information. In this case, the most extensive information available was the data set collected by Cannesson *et al. *[16]. The caveat, of course, is that if the data are erroneous, the closed loop may nevertheless function in the artificial simulation environment because it is partially based on those same data. The third phase of the study was intended in part to examine this possible bias in the design by making the reported PPV an imprecise predictor to see what impact, if any, this had on the LIR algorithm. As demonstrated, the controller continues to function even when PPV varies widely from the "true" value generated from the incorporation of the database into the simulator. Although this suggests that the appropriate operation of LIR is not dependent on the included PPV dataset alone, only further study independent of the simulator will adequately address this concern.

Good evidence indicates that maximizing SV in the perioperative period will reduce complications and improve outcomes for moderate- to high-risk surgical patients [19-24]. Thermodilution with a pulmonary artery catheter remains the clinical standard, but alternative CO monitoring devices (like esophageal Doppler) have also been used to guide therapy with positive effects [25]. With the proliferation of noninvasive and minimally invasive CO devices in recent years, goal-directed CO monitoring is readily achievable in a broad patient population. Hemodynamic management of this type requires frequent interventions, protocol-driven decision making, and high levels of attention to be effective and repeatable. Given these requirements, it may be difficult to gain acceptance for standardized protocols. The more complex a protocol becomes, the harder it is to implement correctly and consistently, and even in the best of cases, errors in implementation are common because of care-provider time constraints [26]. Additionally, health care providers work in an environment full of distractions [27] and are susceptible to fatigue, lapses in attention, and stress, all of which can have a negative impact on work performance [28]. Furthermore, studies have shown that when management protocols are implemented in clinical care, adherence rates are only 40% to 50%, and this remains true across a variety of disciplines from ICU glucose control to trauma assessment and even outpatient screening guidelines [26,29-32].

Closed-loop systems are a bridge across this implementation gap, allowing the closed loop to perform the often tedious tasks of the protocols and intervening when needed, which then allows clinical care providers to focus on other aspects of management. Moreover, the closed loop is not susceptible to distraction, fatigue, or personal bias. This means that a clinical protocol or algorithm can be followed exactly and improved on over time. Once a closed loop is shown to be effective in management of a given clinical scenario, it can be used repeatedly and will produce consistent results in that scenario.

A great deal of automation is already being integrated into clinical care. Closed-loop ventilators that adapt breath-to-breath in response to changes in oxygenation and lung compliance are commonplace in modern ICUs [3,33]. Insulin therapy systems are being actively investigated for both inpatient and outpatient use [34]. A closed-loop propofol and remifentanil administration system based on BIS (processed EEG) was recently reported on in a large clinical trial [35]. Closed loops like these are demonstrating the possible uses and benefits of automation in medicine.

### Study limitations

Although the LIR algorithm proved effective in management of the simulated hemorrhage scenarios, some limitations exist with the current study. First, the simulator was designed with as accurate a hemodynamic model as possible, given the current understanding of the dynamic predictors of fluid responsiveness, but generalizations about the possible efficacy of this controller in clinical practice would be premature based on this initial work. In terms of the algorithm design, the controller has not been rigorously studied for stability and robustness from an engineering standpoint and will need this testing before clinical studies can commence. The patient database used to seed the population-based algorithm is our own, which may limit applicability. Finally, the third phase of our study (in which the tolerance of the algorithm for error in the PPV signal was examined) suggests that the algorithm will still function well in the face of moderate levels of noise and error, but whether this is sufficient to tolerate the noise and uncertainty actually present in clinical monitoring of patients remains to be tested.

A limitation in the comparison with practitioners is the obvious difference between simulation and clinical care. It may well be that clinical cues and observations are available to practitioners in the OR or ICU setting, not available in simulation, that may limit the applicability of the second phase of our study to true clinical practice. Only further testing and clinical trials can adequately address this issue.

Additionally, the decision specifically to exclude blood-product administration may have limited the application of these simulation results to real-world hemorrhage and resuscitation. As previously mentioned, LIR was designed to determine the appropriate volumes for resuscitation, but not the appropriate fluid to give in a particular situation. This decision would be left to the supervising physician, who could hang any fluids or products desired and allow the algorithm to determine the rate and timings of administrations. As this was not a part of the algorithm, we sought to limit the study to the question of volumetric appropriateness only by removing the option of blood products from both groups. A system like LIR would never be deployed in the absence of some form of direct supervision for exactly this reason. The supervising physician would need to determine when blood products were needed; otherwise, in a slow but steady hemorrhage, the controller would continue to give fluids to maintain intravascular volume until the patient died of anemia. A supervising physician would recognize the need for products and could hang them instead of a fluid bag when appropriate. An "anemia risk" alarm could be integrated that tracked parameters like volume given, time span, and patient weight, but this would only be an adjunct to supervision. Moreover, the ongoing development and improvement of continuous, noninvasive hemoglobin sensors represents another possible safety feature and enhancement for future integration [36].

Because of the nature of the scenarios, we limited the patient parameter randomization process in the simulator to narrower ranges than might be found in the real patient population. The reason for this was to keep the scenarios more consistent in their evolution (for example, a 2,000-ml blood loss would have been catastrophic in a 45-kg patient versus a 100-kg patient). This kept the scenarios more consistent across trials, but the restricted range may limit applicability of the efficacy of the LIR algorithm to a large and variable patient population.

Finally, this study did not seek to answer a host of questions regarding this system. For example, how would the system cope with a significant change in patient condition like the onset of atrial fibrillation, or a change from volume control to assisted or spontaneous ventilation? What about a new-onset myocardial infarction? The LIR algorithm was designed to detect when patient responses are not matched by expectations, especially when a sudden change in responses or overall patient condition occurs, and adjust future interventions accordingly, but clearly this is out of the scope of the current study and requires much more testing. Further studies testing the ability of the system to work by using SV optimization alone or PPV/SVV optimization alone are needed.

## Conclusion

The current study demonstrates that the LIR algorithm effectively resuscitates simulated patients in the tested scenarios and significantly improved physician management in the second phase of the study. Future studies will focus on the stability of the controller and testing in a broader range of scenarios, including in other simulators.

## Key Messages

• This study is the first to describe an automated system for hemodynamic optimization based on cardiac output and pulse-pressure variation optimization. These data demonstrate that the learning intravenous resuscitator (LIR) is an effective volumetric resuscitator in simulated hemorrhage scenarios and improved physician management.

• The controller performs comparably to practitioners in this setting and maintains a higher and more stable cardiac output

• The controller is not dependent on a highly predictive pulse-pressure variation value to function

• This system is designed to optimize hemodynamics based on pulse-pressure variation and/or stroke-volume variation and/or stroke volume alone

• Engineering testing and simulation studies are the first required steps before any testing of a closed-loop system in a living system

## Abbreviations

CO: cardiac output; DBP: diastolic blood pressure HR: heart rate; LIR: Learning Intravenous Resuscitator; MAP: mean arterial pressure; ΔPOP: respiratory variations in the plethysmographic waveform amplitude; PPV: pulse-pressure variation; SBP: systolic blood pressure; SVV: stroke-volume variation.

## Competing interests

Maxime Cannesson and Joseph Rinehart are co-inventors and co-owners of US patent serial no. 61/432,081 for a closed-loop fluid-administration system based on the dynamic predictors of fluid responsiveness. Maxime Cannesson is a consultant for Edwards LifeSciences (Irvine, CA, USA), Covidien (Boulder, CO, USA), Masimo Corp. (Irvine, CA, USA), ConMed (Irvine, CA, USA), Philips Medical System (Suresnes, France), CNsystem (Vienna, Austria), BMeye (Amsterdam, Netherlands), and Fresenius Kabi (Sèvres, France). Yannick Le Manach is a consultant for Air Liquide Santé (Paris, France) and received lectures/travel fees from Masimo Corp. (Irvine, CA, USA) and Fresenius Kabi (Sèvres, France). Chris Hofer is a consultant for Pulsion Medical Systems (Munchen, Germany), Edwards LifeSciences (Irvine, CA, USA), CSL Behring (King of Prussia, PA, USA). Benoit Tavernier received lectures/travel fees from Masimo Corp. (Irvine, CA, USA) and Fresenius Kabi (Sèvres, France).

## Authors' contributions

JR designed the controller and the simulator, designed the study, collected and analyzed the data, drafted the manuscript, and gave final approval of the manuscript. BA designed the controller and the simulator, drafted the manuscript, and gave final approval of the manuscript. YLM, CH, BT, and ZNK participated in data analysis and interpretation, drafted the manuscript, and gave final approval of the manuscript. MC designed the controller and the simulator, designed and coordinated the study, analyzed the data, drafted the manuscript, and gave final approval of the manuscript.

## Supplementary Material

Additional file 1 **Hemodynamic Simulator Design**. This document describes how the hemodynamic simulator was design. It provides an in-depth description of the mathematical and physiological models used to build the hemodynamic simulator used in the present study. Click here for file
