# Supplementary material for: Evaluation of a novel closed-loop fluid-administration system based on dynamic predictors of fluid responsiveness: an in silico simulation study
Source: Crit Care. 2011 Nov 23;15(6):R278. doi: 10.1186/cc10562 (PMC3388660; doi:10.1186/cc10562)
Supplement: Additional file 1 — Hemodynamic Simulator Design. This document describes how the hemodynamic simulator was design. It provides an in-depth description of the mathematical and physiological models used to build the hemodynamic simulator used in the present study. [file cc10562-S1.PDF]

Appendix 1: Design of the Hemodynamic Simulator

Introduction

The first objective was to define the purpose of the simulator to guide the design specifications. Our goal was to investigate acute resuscitation in a variety of hemorrhage scenarios, so we needed a simulator capable of accurately modeling heart rate (HR), systolic blood pressure (SBP), diastolic blood pressure (DBP), cardiac output (CO), and one or more dynamic parameter of fluid responsiveness (e.g. pulse pressure variation (PPV), systolic pressure variation (SVV), or plethysmograph variability (DeltaPOP) over a wide range of blood volumes. A system designed with model elements specifically based on anatomic counterparts was a natural approach to this problem; our model would therefore include the base components of the cardiovascular system: the heart, arterial vascular tree, end organ capillaries, and the venous tree. Each of these components was designed as a distinct unit with specific inputs and outputs to the other units in anatomic sequence. As our simulator was intended to represent otherwise healthy patients, all components were modeled to behave as they would in the absence of any organ pathology. Like most simulation models, the elements are simplifications of the true physiology. Despite these simplifications, however, we aimed to faithfully preserve the relationships between the elements themselves and the final simulator outputs. Widely accepted physiological laws such as those described by Guyton would be used wherever possible. Especially important would be relationships between cardiac output, mean arterial pressure, and blood volume changes during hemorrhage [1, 2]. Regarding the evolution of dynamic parameters of fluid responsiveness and their relationship with hemorrhage and fluid responsiveness status, we used previously published studies on the topic [3, 4] as well as our own data [5].

Methods

The model began with a baseline state that included a height in inches (in), weight in kilos (Kg), HR, SBP, DBP, central venous pressure (CVP), left ventricular end-diastolic volume (LVEDV), and left-ventricular end-systolic volume (LVESV). These parameters were randomized within pre-determined ranges, and from these starting values, resulting baseline values for other parameters were calculated (Table 1). Additional parameters that were determined in advance were: PPV wander (PPV_W), an error range through which PPV wandered throughout the simulation at random; and PPV error (PPV_E), a constant positive or negative error added to PPV which did not change. Two additional hidden & randomized parameters were Patient Stability (P_S) and Patient Fluctuation (P_F). Patient Stability influenced how rapidly the patient physiology changed in response to stress or stimulus; a higher stability made the patient slower to adapt to hypotension, for example, and might reflect old age. Patient Fluctuation determined how stable the patient was in the absence of stress or stimulus. Higher values increased small and random minute to minute changes in HR, MAP, and CO. These values and the other equation variables found below, along with their typical ranges, are also listed in Table 1.

Beginning with the venous tree, venous return to the heart (in the form of left ventricular preload) was dependent only on the current blood volume relative to the baseline blood volume. The right ventricle and pulmonary circuit were assumed to be healthy and therefore considered passive elements in this design (any volume delivered to the right atria was delivered without interference to the left atria). The effect of blood volume on LVEDV needed to describe a curve wherein a 10% blood loss had virtually no effect on hemodynamics and a 40-45% blood loss was fatal, consistent with clinical and experimental findings[1-4]. The equation used to determine LVEDV at time *t*, then, was:

$$L_t = LVEDV_{t-1} + \frac{(LVEDV_0 * \sin(1.3v + 0.2) \ - LVEDV_{t-1})}{S}$$

where $LVEDV_O$ is the LVEDV at baseline, $LVEDV_{t-1}$ is the LVEDV for the preceding cardiac cycle, *v* is the ratio of current blood volume divided by baseline blood volume, and *S* is a patient constant determining global hemodynamic reactivity. This function describes the curve shown in Figure 1. *P* was further decreased by 1% for each 2 beats per minute the heart rate exceeded 100 to reflect decreased ventricular filling time. This volume was then passed into the heart element.

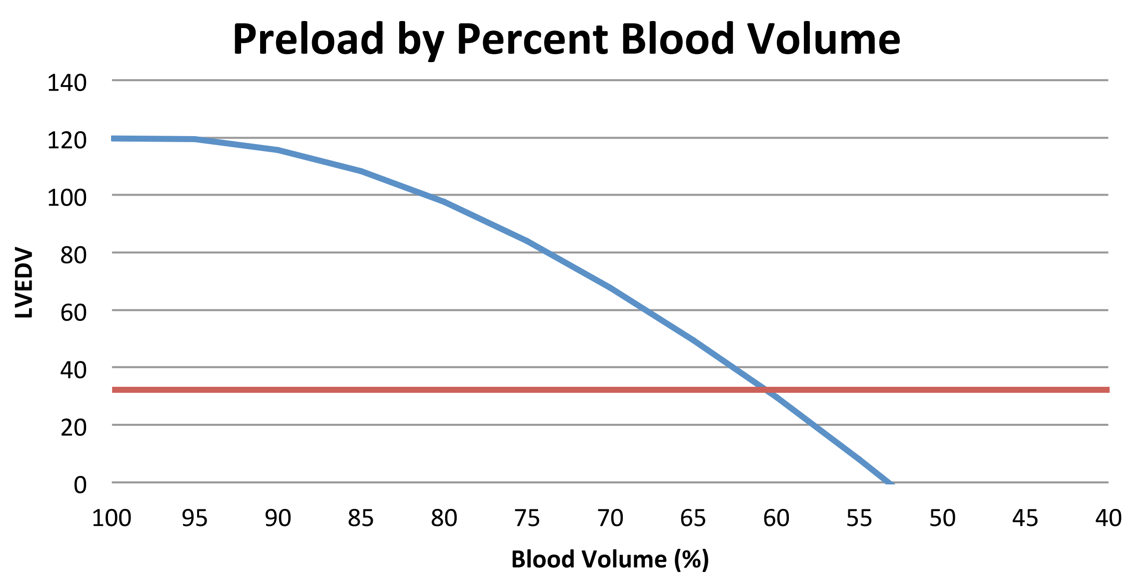

The red line indicates the average limit of survivability in the simulator model. Below this volume level there is virtually no cardiac output.

Figure 1: Simulated Left Ventricular End Diastolic Volume by Percent Blood Volume

Once the heart received the LVEDV value, a stroke volume needed to be determined. This was accomplished by using the baseline LVESV and calculating changes that would be expected in the current physiologic state. The end result would be a stroke volume that needed to reflect true physiologic responses. In addition to changes due to LVEDV increase or decrease, SV needed to decrease in the presence of high afterload, and to increase from sympathetic stimulation. The equation used, then, to determine SV at time *t* was:

$$SV_t = LVEDV_t - \left(\frac{(3LVESV_{t-1} + LVESV_0)}{4} - 7\Upsilon\right)$$

Where $LVEDV_t$ is the LVEDV at time *t*, $LVESV_0$ is the LVESV at baseline, $LVESV_{t-1}$ is the LVESV during the previous cycle, and ϒ is a measure of global sympathetic activity (often zero, unless MAP or CO have fallen to 25% or more from baseline, or in the presence of inotropes). Additionally, SV could not be less than zero, and would always be at least 15% of the LVEDV regardless of how low the LVEDV became.

The baseline CO determined during the initialization of the simulator run was taken as the ideal value for the patient for the rest of the run. During simulation, HR varied inversely with MAP to reflect baroreceptor activity, was increased by sympathetic activity and sympathomimetics, and was reduced by narcotics (reflecting decreased sympathetic stimulation). The net equation for HR at time *t* was:

$$HR_t = \frac{\left(HR_0\left(\frac{\Upsilon}{6} - \frac{\mu}{25} + 0.4\right) + 0.6HR_{t-1} + \frac{(MAP_0 - MAP_t)}{3.75} - \frac{\mu}{7.5} - I\right)}{S}$$

Where HR_0 is the base heart rate, μ is a measure of opioid activity, ϒ is a measure of global sympathetic and sympathomimetic drug activity (ephedrine or epinephrine, for example), CO_0 and CO_t are baseline and current cardiac output, MAP_0 and MAP_t are the baseline and current mean arterial pressure, *S* is a patient constant determining hemodynamic responsiveness, and *I* is a measure of myocardial ischemia. *I* was zero unless heart rate exceeded MAP by a factor of 6 or more, in which case it became negative in straight proportion to the difference. This overall relationship placed an upper limit on cardiac function of around 160-180 beats per minute, but hypotension would reduce this maximum as ischemia set in.

The final core element in the model was the arterial tree and systemic vascular resistance. In clinical settings, CO and MAP are often used to determine what the SVR is through the simple mathematical relationship CO*SVR/80 = MAP. In the cardiovascular system, however, it is actually the cardiac output and the systemic vascular resistance that dictate what the MAP is, and this is how the simulator was designed to function as well. Baseline systemic vascular resistance (SVR) was determined mathematically at the beginning of the simulation depending on the HR and MAP at that time (Table 1). CVP was assumed to be 5 at baseline and rose or fell by 1 for each 10% change in blood volume for the purposes of SVR calculation. From this baseline SVR rose or fell inversely with MAP in an attempt to maintain perfusion pressure. Additionally, vasopressors and sympathetic tone directly increased SVR and narcotics reduced it. The net equation for SVR at time *t* was:

$$SVR_t = \frac{\left(SVR_{t-1}\left(\frac{MAP_0 + \Upsilon - \mu}{MAP_0} + 0.26\right) + SVR_0(\Upsilon + \alpha + 0.74)\right)}{S}$$

where SVR_{t-1} and SVR_0 is the systemic vascular resistance during the previous cycle and at baseline, MAP_0 is the baseline mean arterial pressure, Υ is a measure of global sympathetic activity, μ is a measure of opioid activity, α is a measure of vasopressor activity, and S is a patient constant determining global hemodynamic reactivity. This value was dampened as it approached physiologic extremes of SVR range (less than 400-600 and greater 2200-2400). Cardiac output is not directly included in this equation; a fall in cardiac output would result in a fall in MAP for any given SVR, and *it is the fall in MAP* that the SVR will respond to in this model.

After the above values were calculated, MAP was determined simply as CO*SVR/80. Systolic and diastolic blood pressures were determined in the same ratio *R* as the initial systolic and diastolic blood pressure:

$$DBP_t = \left(\frac{3MAP_t}{R+2}\right)$$

and

$$SBP_t = R * DBP_t$$

PPV modeling based on true physiologic and anatomic activity would have required thoracic pressures, lung compliance, tidal volume, and more. As the specific interactions between tidal volume, thoracic pressure, and PPV were not the intended focus of this simulator, we chose to simplify the determination while attempting to preserve the relationship of PPV to intravascular volume. In the true physiology, cyclic changes in thoracic pressure cause preload to the heart to increase and decrease over the course of a respiratory cycle. If the patient is on the plateau portion of the Starling Curve this fluctuation has little impact on the stroke volume and pulse pressure of the resultant arterial pressure waveform. On the other hand, if the patient is on the steep portion of the curve the preload fluctuations *do* impact the waveform, and the variation can be measured in stroke volume variability (SVV) or pulse pressure variability (PPV).

Knowing that under the appropriate conditions[5] the known (measured) PPV can predict the increase in CO expected in response to a fluid bolus, we designed the simulator to work backwards from the real world[6, 7]. As it was easy to calculate the impact that additional vascular volume would have in our model, the known expected CO increase is used to determine the unknown PPV. First, the change in cardiac output that a 500ml fluid bolus would create is calculated:

$$\Delta CO = \frac{\left(HR_t * (SV_{500} - SV_t)\right)}{1000}$$

Where ΔCO is the cardiac output if there was 500ml additional volume in the vascular tree, SV_{500} is the stroke volume with 500ml additional volume, and SV_t is the current real stroke volume. Once the ΔCO that additional volume would generate was determined, this value was taken to the PPV database described in the text [5] and the mean PPV value

which predicted this increase in similar HR & MAP ranges was found and output by the simulator.

Finally, loss of crystalloid from the vascular tree occurred at a rate of 60% per hour for crystalloids and 20% per hour for colloids[8]. Of note, the model did not include the effect of airway pressure changes on PPV, consideration of patient age or health problems, or blood viscosity changes that would occur during hemorrhage and resuscitation.

Data was displayed on a monitor that was configured similarly to the monitors used clinically for anesthesia & critical care (Figure 2). Waveforms were generated based on the underlying physiologic data, including variability of arterial and plethysmograph waveforms where appropriate.

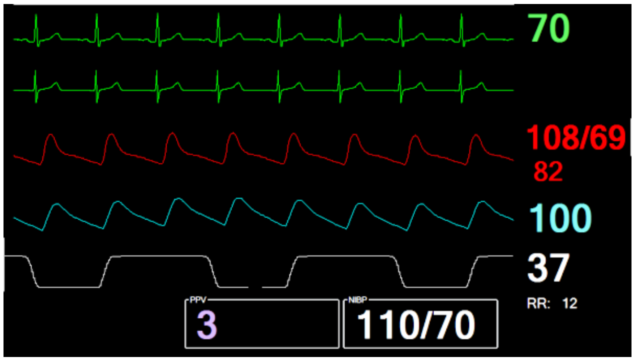

Simulator Display Screen at Sample Patient Baseline

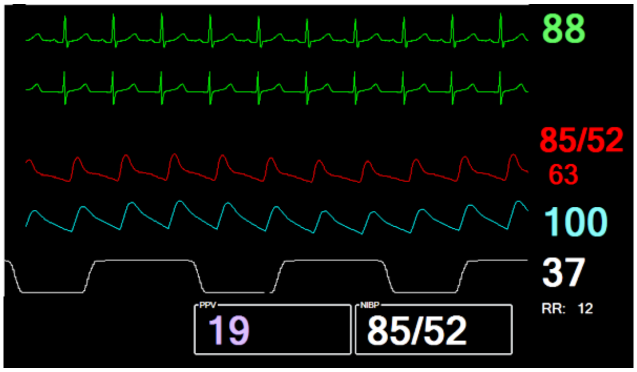

Simulator display screen after hemorrhage showing arterial line and plethysmographic waveform variability

Figure 2. *Simulator display examples*

Validation of the Model

Figure 3 shows the impact of a decrease in blood volume on CO and MAP simulated by the system. The evolution in these parameters exhibits the evolution described by Guyton with a MAP held constant until a 20 % decrease in blood volume is reached while CO starts decreasing earlier, as soon as a ~10 % decrease in blood volume is reached. Both curves rapidly approach zero around 40% decrease in blood volume, also consistent with previously described hemorrhage models [2].

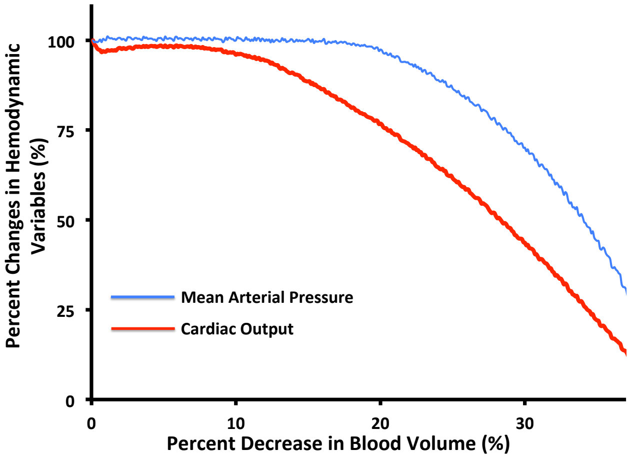

Figure 3. Evolution in mean arterial pressure and cardiac output relative to changes in blood volume.

Figure 4 shows the impact of a decrease in blood volume on HR, LVEDV, CO, MAP, SVR, and PPV simulated by the system. As described here, despite an early decrease in

CO, MAP is regulated initially by an increase in SVR. The relationship between LVDEV and blood volume is almost linear so that the decrease in CO observed initially is related to a decrease in LVEDV rather than a decrease in HR. This figure and its results models data obtained previously in human and animal studies [3, 4].

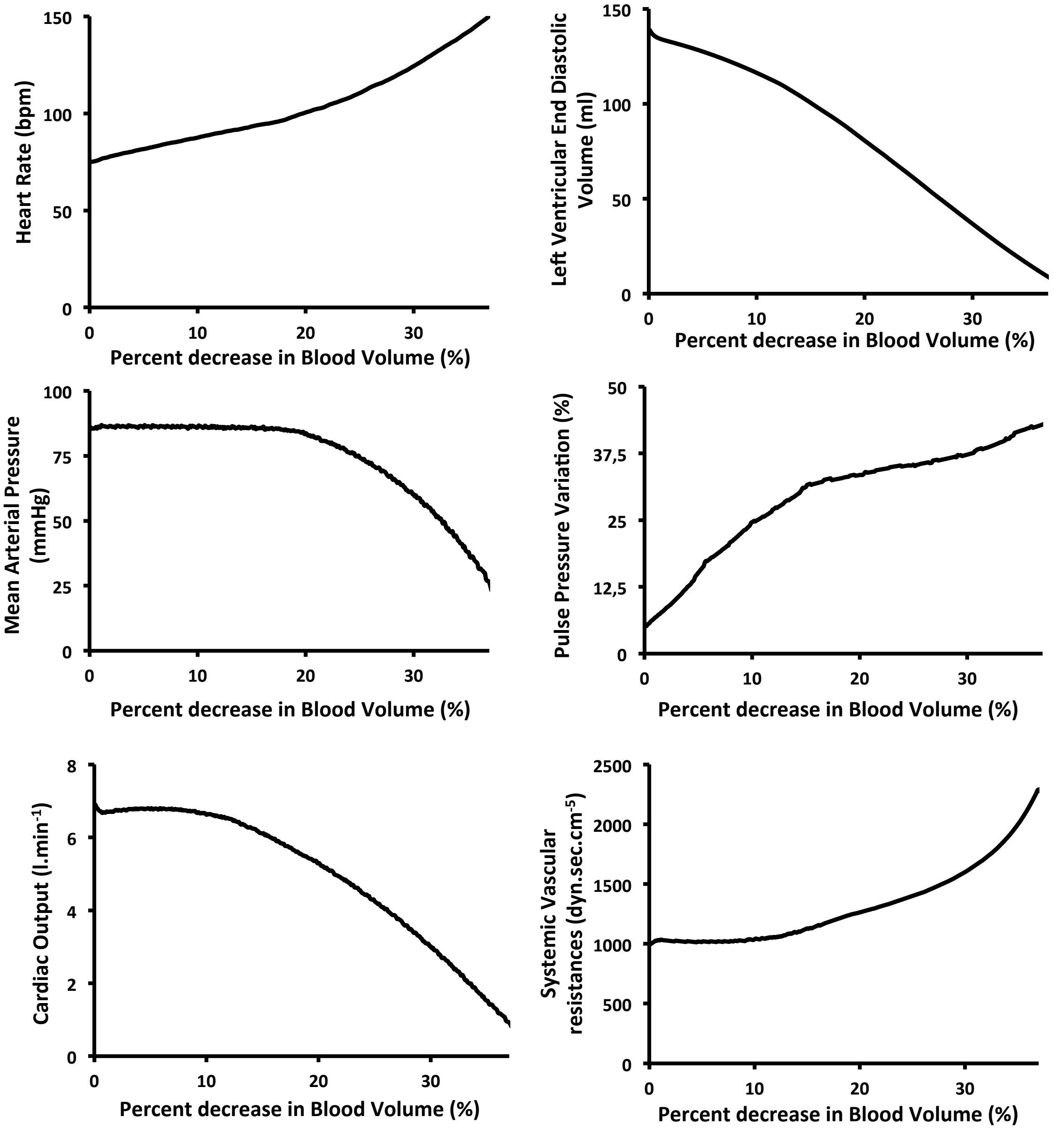

Figure 3. Simulated changes in hemodynamic variables induced by a decrease in blood volume.

References

1. Guyton AH, Hall JE: **Heart Muscle; The heart as a pump and function of the heart valves.** In: *Textbook of medical physiology, 11th edition.* edn. Edited by Elsevier S. Philadelphia: Elsevier, Inc; 2006: 103-115.
2. Guyton AH, Hall JE: **Overview of the circulation: medical physics of pressure, flow, and resistance.** In: *Textbook of medical physiology, 11th edition.* edn. Edited by Elsevier S. Philadelphia: Elsevier, Inc; 2006: 161-170.
3. Perel A, Pizov R, Cotev S: **Systolic blood pressure variation is a sensitive indicator of hypovolemia in ventilated dogs subjected to graded hemorrhage**. *Anesthesiology* 1987, **67**(4):498-502.
4. Pizov R, Eden A, Bystritski D, Kalina E, Tamir A, Gelman S: **Arterial and plethysmographic waveform analysis in anesthetized patients with hypovolemia**. *Anesthesiology* 2010, **113**(1):83-91.
5. Cannesson M, Le Manach Y, Hofer CK, Goarin JP, Lehot JJ, Vallet B, Tavernier B: **Assessing the Diagnostic Accuracy of Pulse Pressure Variations for the Prediction of Fluid Responsiveness: a "Gray Zone" Approach**. *Anesthesiology* 2011, **(In Press)**.
6. Schbeeb A, Alexander B, Rinehart J, Cannesson M: **The Use of Pulse Pressure Variation in Predicting Fluid Responsiveness**. *Anesthesiology* 2011, **In Press**:A1650.
7. Marik PE, Cavallazzi R, Vasu T, Hirani A: **Dynamic changes in arterial waveform derived variables and fluid responsiveness in mechanically ventilated patients: a systematic review of the literature.** *Crit Care Med* 2009, **37**:2642-2647.
8. Greenfield RH, Bessen HA, Henneman PL: **Effect of crystalloid infusion on hematocrit and intravascular volume in healthy, nonbleeding subjects**. *Annals of emergency medicine* 1989, **18**(1):51-55.

Table 1: Simulator Variables, Ranges, and Determinations

Symbol	Parameter	Typical Range	Baseline Randomized	Calculation
in	Height	60-72	Yes	
Kg	Weight	60-85	Yes	
HR	Heart Rate	55-85	Yes	
CVP	Central Venous Pressure	0-10	Yes	
LVEDV	Left Ventricular End Diastolic Volume	130-150	Yes	
LVESV	Left Ventricular End Systolic Volume	42-58	Yes	
SBP	Systolic Blood Pressure	110-130	Yes	
DBP	Diastolic Blood Pressure	60-80	Yes	
MAP	Mean Arterial Pressure	80-90	No	(2*DBP + SBP)/3
R	Systolic-Diastolic Ratio	3:2	No	SBP / DBP
SV	Stroke Volume	80-100	No	LVEDV - LVESV
CO	Cardiac Output	6.0-8.0	No	(HR * SV) / 1000
V_0	Ideal Blood Volume in Liters	4.2 - 5.9	No	Kg * 70
SVR	Systemic Vascular Resistance	900-1200	No	80*(MAP-CVP)/CO
P_S	Patient Stability	30-160	Yes	
P_F	Patient Fluctuation	0-2	Yes	
PPV_W	PPV Wander	0-10	Dependent[2]	
PPV_E	PPV Error	0-5	Dependent[2]	
Υ	Total Sympatehtic Effect	0-2[1]	No	
α	Vasopressor activity	0-2,000	No	
μ	Opioid activity	0-10	No	
R	Systolic-Diastolic Ratio	1.2-1.8	No	
I	Myocardial Ischemia	0-20	No	

1 -In extremely low MAP/CO conditions, or as a result of large epinephrine doses, ϒ may have exceeded this range, but for all study test conditions ϒ fell within these bounds.

2 - These values were constant in some scenarios and randomized in others as described in the primary text
